# The impact of emergency department segmentation and nursing staffing increase on inpatient mortality and management times

**DOI:** 10.1186/s12913-016-1544-x

**Published:** 2016-07-19

**Authors:** Pierre-Géraud Claret, Xavier Bobbia, Sylvia Olive, Christophe Demattei, Justin Yan, Robert Cohendy, Paul Landais, Jean Emmanuel de la Coussaye

**Affiliations:** Department of Anesthesia Resuscitation Pain Emergency Medicine, Nîmes University Hospital, 4 Rue du Professeur Robert Debré, 30029 Nîmes, France; EA 2415, Clinical Research University Institute, Montpellier University, 641 Avenue du Doyen Gaston Giraud, 34093 Montpellier, France; Department of Biostatistics, Clinical Research, Clinical Epidemiology, and Public Health, Nîmes University Hospital, 4 Rue du Professeur Robert Debré, 30029 Nîmes, France; Division of Emergency Medicine, Department of Medicine, London Health Sciences Centre and The Schulich School of Medicine and Dentistry, The University of Western Ontario, London, ON Canada; Montpellier-Nîmes University, 2 rue École de Médecine, 34060 Montpellier, France

**Keywords:** Emergency service, Hospital, Organizations, Nursing staff

## Abstract

**Background:**

The aim of our study was to investigate the impact of a new organization of our emergency department (ED) on patients’ mortality and management delays.

**Methods:**

The ED segmentation consisted of the development of a new patient care geographical layout on a pre-existing site and changing the organization of patient flow. It took place on May 10, 2012. We did a before-after study in the ED of a university hospital, “before” (winter 2012) and “after” (summer 2012) reorganization by segmentation into sectors. All ED patients were included.

**Results:**

Eighty-three thousand three hundred twenty-two patient visits were analyzed, 61,118 in phase “before”, 22,204 during the phase “after”. The overall inpatient mortality was 1.5 % during summer 2011 (“before” period), 1.8 % during winter 2012 (“before” period), 1.3 % during summer 2012 (“after” period) period (summer 2012 vs. winter 2012, OR = 0.72; 95 % CIs [0.61, 0.85], and summer 2012 vs. summer 2011, OR = 0.85; 95 % CIs [0.72, 0.99]). The mean (SD) time to first medical contact was 129 min (±133) during winter 2012 and 104 min (± 95) during summer 2012 (*p* < .05).

**Conclusions:**

Our study showed a decrease in mortality and improvement in time to first medical contact after the segmentation of our ED and nursing staffing increase, without an increase in medical personnel. Improving patient care through optimizing ED segmentation may be an effective strategy.

## Background

Overcrowding in emergency departments (EDs) has adverse consequences for patients such as delayed care [1], decreased patient safety [2], and increased morbidity and mortality [3]. The causes of overcrowding are multiple and intertwined, but increased delays in care seem to have a major impact on ED flow [3, 4]. To analyze this issue, important variables to consider include the delay to first medical contact (FMC) [5] and the length of stay (LOS) in the ED [3]. These times are recognized as indicators of overcrowding and can be considered targets on which to act to improve outcomes for ED patients [6].

Previous studies have reported on specific solutions regarding the internal organization of EDs and the impact on reducing delays to FMC and LOS, and thus overcrowding [6, 7]. However, the effect of ED organization with operational teams working in separate architectural entities on patient mortality has not been described.

Our ED was reorganized and segmented into sectors. Before segmentation, there was no allocation of patients to a specific physician or nurse. The segmentation was defined as a new organization of the caregivers into several sectors corresponding to the functional and architectural entities. Our objective was to investigate the impact of this new organization. Our primary outcome was the inpatient all-cause mortality rate of all patients admitted from the ED. Our hypothesis was that organization by sectors decreases inpatient mortality and ED delays.

## Methods

### Study setting and population

This study was conducted in the ED of a university hospital, in Nîmes, France, with an average annual census of 75,000 visits per year. The ED welcomes all medical and surgical emergencies for adults and children, except ophthalmological and gynecological emergencies. Our hospital consists of an 870-bed (medicine, surgery, and obstetrics), university-affiliated tertiary care hospital with 76,000 inpatient admissions and 317,000 ambulatory visits per year. We included all ED patient visits in our study.

### Study design

**Fig. 1:**
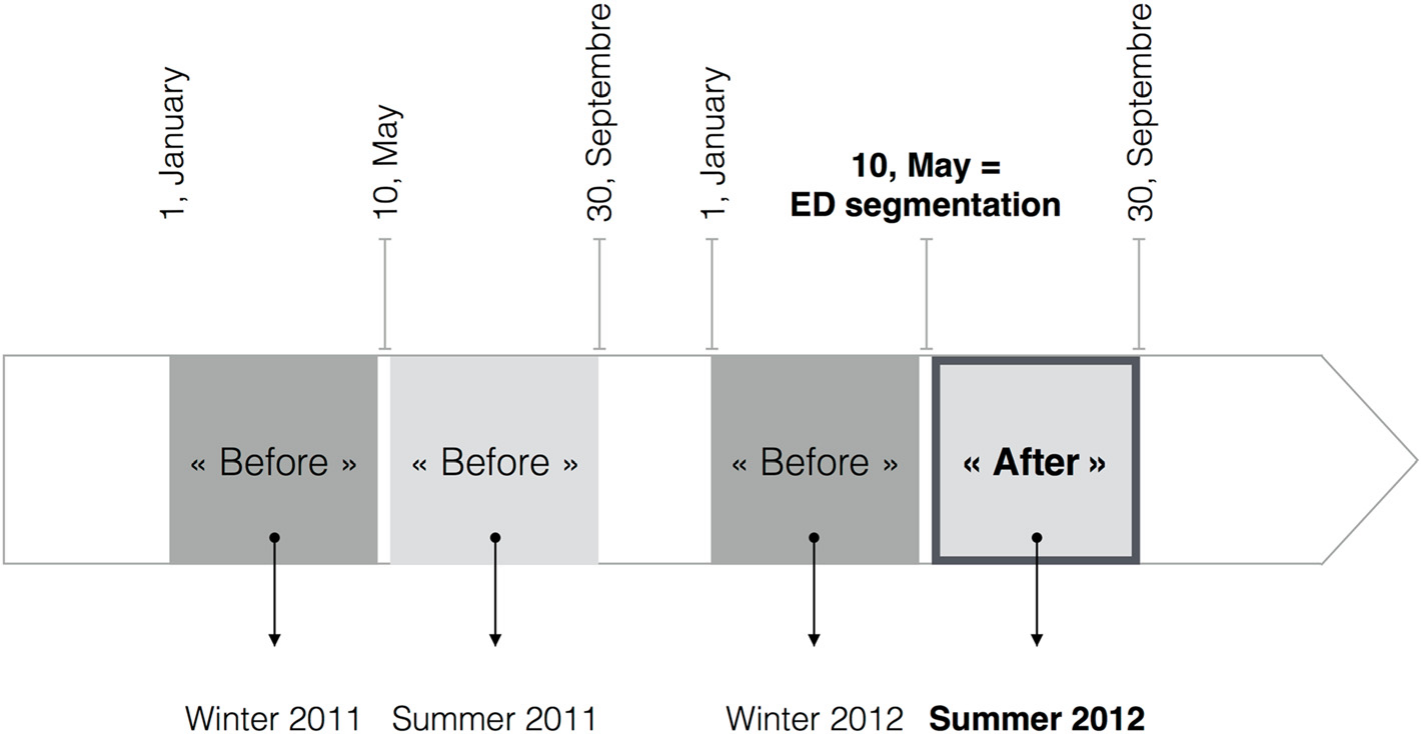
Study design, “before” and “after” periods

We conducted a “before-after” study of segmentation of the ED including all patient visits to the ED from January 1, 2011 to September 30, 2011 and from January 1, 2012 to September 30, 2012. The ED segmentation consisted of the development of a new patient care geographical layout on a pre-existing site and changing the organization of patient flow. It took place on May 10, 2012. All patient visits to the ED between January 1, 2011 and the implementation of the organizational change were considered as belonging to the “before” period, the others to the “after” period. Winter was defined as the period from January 1 to May 10. Summer was defined as the period from May 10 to September 30. Therefore, the “before” periods included winter 2011, summer 2011, and winter 2012. The “after” period was defined as summer 2012 (Fig. 1). This study was approved by the Institutional Review Board of Nîmes University Hospital and was declared to the National Commission for Data Processing and Civil Liberties.

### Emergency department segmentation

**Fig. 2:**
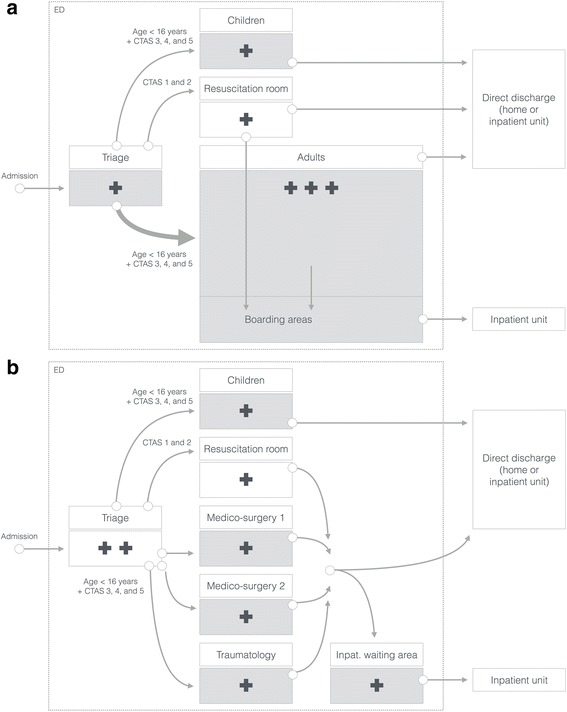
Comparison of ED organization before (a) and after (b) ED segmentation. Sector in green: sector led by a physician, red cross: nurse

Entities “before” segmentation included the triage unit (one physician, one nurse) using the Canadian Triage and Acuity Scale (CTAS), pediatric emergencies (one physician, one resident, one nurse), and a medico-surgical unit for adults (three physicians, four residents, three nurses) where most of the patients were admitted. The medico-surgical unit included a resuscitation room for life-threatening emergencies (CTAS 1 and 2). Before segmentation, there was no allocation of patients to a specific physician or nurse. Patients who would have been admitted to a ward if a bed was available, waited most of the time in the ED hallways (Fig. 2–a). The segmentation was defined as a new organization of the caregivers into seven sectors corresponding to seven architectural entities: the medico-surgical units I and II (one physician, one resident, one nurse), the traumatic emergencies (one physician, one resident, one nurse), the pediatric emergencies (one physician, one resident, one nurse), the inpatient waiting area (one physician, one nurse), the patients’ triage sector (one nurse in the morning, two in the afternoon) and the resuscitation room (one resident, one nurse). The physician responsible for traumatic emergencies also supported the triage nurse if needed, while one of the physicians from the medico-surgical units supported the resuscitation room if needed. The referral to each sector was made by the triage nurse (Fig. 2–b). Segmentation therefore did not require an increase in physician staffing, but required an increase in nurse staffing. Patients who would have been admitted to a ward if a bed was available waited in the inpatient waiting area.

### Measurements

The data were collected through the hospital’s electronic medical record system (InterSystems, Cambridge, United States). The patients were sorted according to CTAS. Inpatient LOS was calculated for hospitalized patients. In our hospital as in many countries, mortality rate is generally higher during the winter and because “before” and “after” periods occurred in different seasons, we analyzed mortality data according to the same time periods the preceding year. Thus, we choose three “before” periods, to adjust for differences in mortality rates across the various seasons.

### Outcomes

Our primary outcome was the inpatient all-cause mortality rate of all patients admitted from the ED. Secondary outcomes were the 24-hour and 30-day inpatient mortality, delays to FMC, ED LOS, inpatient LOS, and triage delays.

### Data analysis

Qualitative variables were compared using a Chi-squared or Fisher exact test. Quantitative variables were compared using Student *t*-test or analysis of variance. In cases of non-parametric distributions, the Wilcoxon-Mann-Whitney test was utilized. The results were presented as means (SD) or medians (IQR) where appropriate. For qualitative variables, numbers and percentages were presented. CTAS 1 times were not analyzed because patients’ management was immediate. All tests were two-tailed and all statistical analyses were carried out in R (www.r-project.org). *P* values below .05 were considered statistically significant.

## Results

### Characteristics of study subjects

Eighty-three thousand three hundred twenty-two patient visits were analyzed, 61,118 in the “before” phase, 22,204 during the “after” phase. During winter and summer 2012, there were 19,017 women (46 %), mean (± SD) age was 39 years (± 28). The triage level distribution was as follows: 354 patient visits (1 %) for CTAS 1; 2864 (7 %) for CTAS 2; 10,026 (24 %) for CTAS 3; and 27,754 (68 %) for both CTAS 4 and 5. These characteristics were not statistically different when comparing the winter (“before” period) and summer 2012 (“after” period). Results are shown in Table 1.

**Table 1:** Characteristics of patients during winter and summer 2012

Variables	Before: winter 2012	After: summer 2012	*p*
	(18,795 patients)	(22,204 patients)	
Fmc, min - mean ± sd (median; iqr)			
overall	129 ± 133 (93; 53–160)	104 ± 95 (80; 49–130)	**
ctas level 2	112 ± 118 (79; 43–145)	69 ± 55 (52; 27–89)	**
ctas level 3	123 ± 132 (94; 53–160)	101 ± 92 (77; 47–128)	**
ctas levels 4 and 5	137 ± 150 (97; 56–165)	107 ± 99 (82; 50–12)	**
Emergency room los, h - mean ± sd (median; iqr)	7 ± 7 (5; 3–9)	6 ± 9 (4; 2–8)	**
Inpatient los, days - mean ± sd (median; iqr)	9 ± 15 (6; 2–11)	8 ± 11 (5; 2–10)	**
Triage delay, min - mean ± sd (median; iqr)	7 ± 33 (1; 0-1)	3 ± 30 (1; 0-1)	**

*Ctas* canadian triage and acuity scale, *ed* emergency department, *fmc* first medical contact, *los* length of stay, “*winter*” 1 January to 10 may, “*summer*” may 10 to September 30, *ns* non-significant, **: *p* < .01

### Main results

**Fig. 3:**
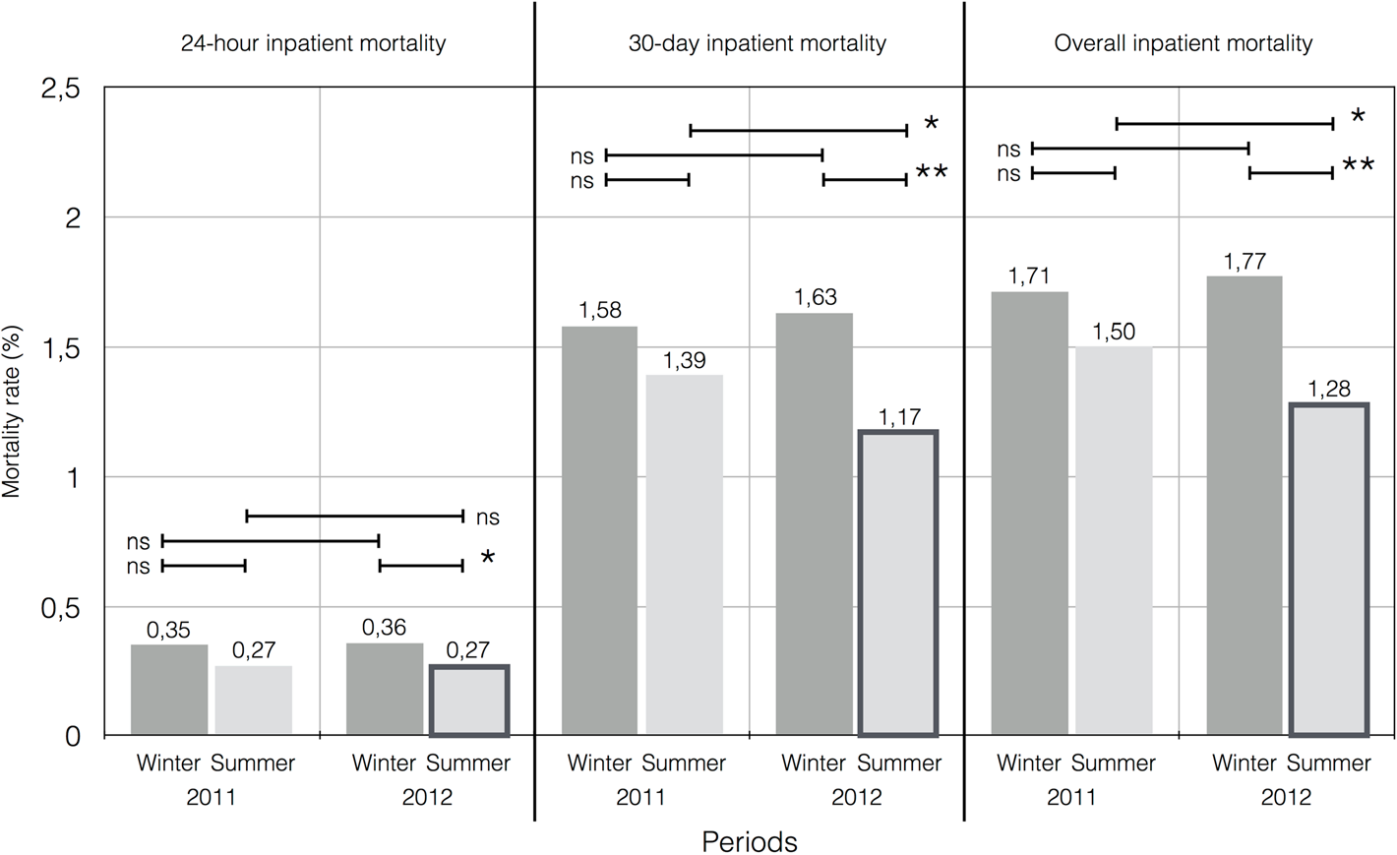
Comparison of mortality of patients admitted to the ED in 2011 and 2012, according to the “before” (winter 2012) and “after” (summer 2012) periods. ns: non-significant, *: *p* < .05, **: *p* < .01

The overall inpatient mortality was 1.5 % during winter 2011 (“before” period), 1.5 % during summer 2011 (“before” period), 1.8 % during winter 2012 (“before” period), 1.3 % during summer 2012 (“after” period) period (summer 2012 vs. winter 2012, OR = 0.72; 95 % CIs [0.61, 0.85], and summer 2012 vs. summer 2011, OR = 0.85; 95 % CIs [0.72, 0.99]). The mean (SD) time to FMC was 129 min (± 133) during winter 2012 and 104 min (± 95) during summer 2012 (*p* < .05). The mean (SD) emergency room LOS was 7 h (± 7) during winter 2012 and 6 h (± 9) during summer 2012 (*p* < .01). Similarly, the mean (SD) inpatient LOS was 9 days (± 15) during winter 2012 and 8 days (± 11) during summer 2012 (*p* < .01). Triage delays were also reduced after the ED segmentation: the mean (SD) triage delay was 7 min (± 33) during winter 2012 and 3 min (± 33) during summer 2012 (*p* < .01). Results are shown in Table 2 and Fig. 3.

**Table 2:** Comparison of outcomes before and after ED segmentation

Variables	2011				2012			
	Before: winter 2011	Before: summer 2011	*p*	OR	Before: winter 2012	After: summer 2012	*p*	OR
	(19,799 patients)	(22,524 patients)			(18,795 patients)	(22,204 patients)		
24-hours in-hospital mortality - *n* (%)	70 (0.4 %)	62 (0.3 %)	ns	0.78 [0.54–1.11]	84 (0.4 %)	60 (0.3 %)	**	0.60 [0.43–0.85]
30-days in-hospital mortality - *n* (%)	313 (1.6 %)	313 (1.4 %)	ns	0.88 [0.75–1.03]	307 (1.6 %)	259 (1.2 %)	**	0.71 [0.60–0.84]
In-hospital mortality - *n* (%)	340 (1.5 %)	340 (1.5 %)	ns	0.88 [0.75–1.02]	333 (1.8 %)	284 (1.3 %)	**	0.72 [0.61–0.85]

“*Winter*” 1 January to 10 May, “*Summer*” May 10 to September 30, *ns* non significant, **: *p* < .01

## Discussion

### Interpretation

The primary objective of our study was to investigate the impact of our new ED organization on the mortality rate of patients admitted from the ED. Our study shows a significant decrease in inpatient mortality after segmentation and nursing staffing increase, when compared with the same time the year before. This study showed the effectiveness of the segmentation of our ED. The decreases in time to FMC and LOS in the ED were associated with a decrease in mortality. Segmentation of our ED involved many changes: an increase in the number of nurses, creation of an inpatient waiting area, formation of operational teams and architectural segmentation of teams and patients. We assume that all of these changes resulted in the observed efficiency. During the study period, there were no other quality improvement initiatives occurring at the hospital that should have influenced the outcomes.

### Previous literature

Previous studies have shown that a new ED organizational system might contribute to reducing patients’ time delays, overcrowding and therefore mortality [3, 4, 6]. Although an increase in physician staffing generally leads to a reduction in overcrowding [7], this was not the case in our study. Indeed, there was no increase in physician staffing since a physician was removed from the triage area and transferred to the hospitalization waiting area. The removal of the triage physician could have also resulted in the deterioration of support for the most serious patients [8]. However, this was not the case in our study, possibly because the delays at triage remained unchanged. We assume that dedicating a physician to patients waiting for hospitalization contributed to improving time to FMC by allowing other physicians to focus on new ED patients. This physician continued to care for patients according to their evolution while continuing communication with other hospital wards for admission. The increase in the number of nurses [9] also likely contributed to improvements in care. The 24-hour mortality rate was not different when summer 2011 was compared with summer 2012. These results show that organizational improvements, except for triage, have little impact on the recognition of immediate life-threatening emergencies and care for the most severe patients. Segmentation also helped decrease patients’ management delays. Pines et al. [1] showed that overcrowding reduced the delay in prescribing antibiotics to patients treated for pulmonary emergencies. The efficiency requirements needed for patients with pathology involving a medium-term prognosis probably help to explain the relationship between reducing delays in care delivery and reduction of hospital mortality.

### Clinical impact

First, the segmentation of our ED required investments in staffing and ED layout. However, this was justified by the gain in the quality of care. Considering 2011 as the reference, there should have been approximately 340 inpatient deaths during summer 2012 (95 % CIs [301–382]), but in actuality, there were only 284 deaths. The decrease in mortality rate in our study reinforces the idea that our restructuring has led to an improvement in patients’ outcomes. We assume that the reduction in LOS [10] may also have had a positive economic effect. Even though this study is not a health-economics study, the economic benefits of reducing FMC and LOS in the ED may be potentially substantial as they may decrease hospital length of stay.

Second, we assume that creating teams in units is a good solution to modify the way to work. Prior to ED segmentation, the physicians at our site were not assigned to specific sections of the ED. In light of this fact and many patients are waiting to be seen simultaneously, individual ED physicians may not feel any sense of urgency to see the next new patient. Reasons for this mindset are varied: an ED physician may feel that he/she has more patients, has sicker patients, or has picked up the most recent chart. With segmentation of the ED, positive changes might improve the flow of patients: the communication between nurses and physicians may be improved because the nurses know which physician is in charge of a particular patient; the size of each physician’s area of care is smaller, meaning less walking; and the physician now has full responsibility in his/her area [11].

This ED segmentation process is based on Lean techniques which were developed by Toyota in the 1972, and can be adapted to help redesign some aspects of ED such as throughput times and utilization of treatment spaces [12, 13].

### Future research

We have not studied the economic effects of the segmentation. However, an economic analysis of a new organizational model is important to conduct and can strengthen the argument for funding. Secondly, our study is limited because it only analyzed the 5-month period immediately following the implementation of the segmentation. Another study is needed to confirm that the observed effects may have a long-term and enduring impact.

### Limitations

Firstly, our study is single-centered, affecting the generalizability of the results. The segmentation was performed with organizational criteria, but also architectural criteria which may not be easily applied to other EDs. Secondly, the data analysis does not control for patient volume between the study periods. However, this control should not decrease the significance of the analysis since the volume of patients was higher after the new ED organization. Third, although some patients were enrolled more than once, we chose not to adjust the analysis because our unit of interest involved all visits to the ED and not individual unique patients. Finally, although our study is retrospective, the data collection ensured the inclusion of all patients presenting to the ED within the study period.

## Conclusions

Opportunities to improve patient flow in the ED are plentiful, and hospitals that engage staff in an improvement effort can derive substantial benefit in cost, quality of care, and patient satisfaction. The segmentation of our ED has led to changes in nurse staffing and ED structural layout, but was associated with a reduction in overcrowding. While there may be an association, it is difficult to support a statement of causation. Nevertheless, improving patient care through optimizing ED segmentation may be an effective strategy. Because each hospital is different, evaluating which improvements to ED design are relevant should be based on performance benchmarks established in consultation with hospital administration and the ED staff.

## Abbreviations

CTAS, Canadian triage and acuity scale; ED, emergency department; FMC, first medical contact; LOS, length of stay

## References

[1] Pines JM, Hollander JE (2008). Emergency department crowding is associated with poor care for patients with severe pain. Ann Emerg Med.

[2] Kulstad EB, Sikka R, Sweis RT, Kelley KM, Rzechula KH (2010). ED overcrowding is associated with an increased frequency of medication errors. Am J Emerg Med.

[3] Guttmann A, Schull MJ, Vermeulen MJ, Stukel TA (2011). Association between waiting times and short term mortality and hospital admission after departure from emergency department: population based cohort study from Ontario, Canada. BMJ.

[4] Hoot NR, Aronsky D (2008). Systematic review of emergency department crowding: causes, effects, and solutions. Ann Emerg Med.

[5] Claret PG, Sebbanne M, Bobbia X, Bonnec JM, Pommet S, Jebali C (2012). First medical contact and physicians’ opinion after the implementation of an electronic record system. Am J Emerg Med.

[6] Geelhoed GC, de Klerk NH (2012). Emergency department overcrowding, mortality and the 4-hour rule in Western Australia. Med J Aust.

[7] Paul JA, Lin L (2012). Models for improving patient throughput and waiting at hospital emergency departments. J Emerg Med.

[8] Holroyd BR, Bullard MJ, Latoszek K, Gordon D, Allen S, Tam S (2007). Impact of a triage liaison physician on emergency department overcrowding and throughput: a randomized controlled trial. Acad Emerg Med.

[9] Harris A, Sharma A (2010). Access block and overcrowding in emergency departments: an empirical analysis. Emerg Med J.

[10] Bayley MD, Schwartz JS, Shofer FS, Weiner M, Sites FD, Traber KB (2005). The financial burden of emergency department congestion and hospital crowding for chest pain patients awaiting admission. Ann Emerg Med.

[11] Vose C, Reichard C, Pool S, Snyder M, Burmeister D (2014). Using LEAN to improve a segment of emergency department flow. J Nurs Adm.

[12] ED becomes ‘lean’ and cuts LBTC, LOS times. ED Manag 2008; 20:44-45. 18689224

[13] Zilm F, Crane J, Roche KT (2010). New directions in emergency service operations and planning. J Ambul Care Manage.

